# Comprehensive Echocardiographic Assessment in Moderate Aortic Stenosis with Preserved Ejection Fraction Using Two-Dimensional Speckle-Tracking Echocardiography: Association with Functional Capacity

**DOI:** 10.3390/jcm14228065

**Published:** 2025-11-14

**Authors:** Olga Petrovic, Dimitrije Zrnic, Stasa Vidanovic, Ivana Nedeljkovic, Olga Nedeljkovic-Arsenovic, Ana Petkovic, Ruzica Maksimovic, Sanja Stankovic, Marina Ostojic, Ivana Paunovic, Ivana Jovanovic, Milorad Tesic, Ana Uscumlic, Jelena Vratonjic, Goran Stankovic, Danijela Trifunovic-Zamaklar

**Affiliations:** 1 Department of Cardiology, University Clinical Center of Serbia, 11000 Belgrade, Serbia; ivannanedeljkovic@yahoo.com (I.N.); drmarinaostojic@gmail.com (M.O.); ina.paunovic@gmail.com (I.P.); ivana170679@gmail.com (I.J.); anauscumlic@gmail.com (A.U.); j_dudic@hotmail.com (J.V.); gorastan@gmail.com (G.S.); danijelatrif@gmail.com (D.T.-Z.); 2 Faculty of Medicine, University of Belgrade, 11000 Belgrade, Serbia; stasavidanovic99@gmail.com (S.V.); olganedeljkovic@gmail.com (O.N.-A.); ancipetkovic@gmail.com (A.P.); dr.ruzica.maksimovic@gmail.com (R.M.); 3 General Hospital Kikinda, 23300 Kikinda, Serbia; dimizrnic94@gmail.com; 4 Center for Radiology and Magnetic Resonance Imaging, University Clinical Center of Serbia, 11000 Belgrade, Serbia; 5 Diagnostic Department of the Center of Stereotaxic Radiosurgery, Clinic of Neurosurgery, University Clinical Center of Serbia, 11000 Belgrade, Serbia; 6 Centre for Medical Biochemistry, University Clinical Center of Serbia, 11000 Belgrade, Serbia; sanjast2013@gmail.com; 7 Department of Biochemistry, Faculty of Medical Sciences, University of Kragujevac, 34000 Kragujevac, Serbia

**Keywords:** moderate aortic stenosis, cardiopulmonary exercise testing, speckle-tracking echocardiography, left atrial strain, ventilatory efficiency

## Abstract

**Background/Objectives**: Moderate aortic stenosis (AS) with preserved ejection fraction (EF) is common, yet risk stratification remains challenging. Cardiopulmonary exercise testing (CPET) and myocardial mechanics analysis may identify subclinical dysfunction and impaired functional capacity. To evaluate the relationship between functional capacity (by % predicted peak VO_2_), ventilatory efficiency (VE/VCO_2_ slope), and myocardial mechanics (speckle tracking echocardiography—STE), and myocardial work (MW) indices) in moderate AS with preserved EF. **Methods**: We prospectively enrolled 107 patients with moderate AS (AVA 1.0–1.5 cm^2^; mean gradient 20–40 mmHg; EF ≥ 50%). Functional capacity was classified as preserved (≥83% predicted VO_2_) or reduced (<83%). Ventilatory efficiency was defined as good (<30) or poor (≥30) VE/VCO_2_ slope. STE assessed left ventricular (LV), left atrial (LA), and right ventricular (RV) strain, as well as myocardial work indices. **Results**: Patients with reduced % predicted VO_2_ had higher LV end-systolic volume (*p* = 0.035), lower stroke volume index (*p* = 0.020), and smaller indexed aortic valve area (*p* = 0.025), with trends toward lower GLS and myocardial work. In contrast, patients with poor ventilatory efficiency (VE/VCO_2_ ≥ 30) showed significant impairments in global longitudinal strain (GLS, *p* = 0.002), LA reservoir strain (PALS, *p* = 0.019) and LA conduit strain (LA Scd, *p* < 0.001), RV free wall strain (RW FWS, *p* = 0.029), and myocardial work indices (lower GWI and GCW, higher GWW, reduced GWE; all *p* < 0.05). LA Scd emerged as the strongest predictor of poor ventilatory efficiency. (receiver operating characteristic (ROC) area under the curve (AUC) 0.723, 95% confidence interval (CI) 0.623–0.823, *p* < 0.001). **Conclusions**: In moderate AS with preserved EF, impaired ventilatory efficiency is more strongly associated with subclinical LV, LA, and RV dysfunction than reduced % predicted VO_2_, highlighting the key role of RV impairment. Integrating CPET and STE improves phenotyping, identifying high-risk patients who may benefit from closer surveillance or early intervention. These findings are exploratory and hypothesis-generating; longitudinal data are needed to confirm prognostic implications.

## 1. Introduction

Aortic stenosis (AS) is the most common valvular heart disease in developed countries, with moderate AS (aortic valve area (AVA) 1.0–1.5 cm^2^, mean gradient 20–40 mmHg), affecting a significant proportion of elderly patients (estimated prevalence 2–4% in those >65 years) [1,2]. AS is a complex disease affecting the aortic valve, myocardium, coronary microcirculation, and the entire cardiovascular system. Subclinical cardiac dysfunction—manifesting as impaired LV, left atrial (LA), and right ventricular (RV) mechanics—often precedes overt symptoms or ejection fraction (EF) decline, highlighting the need for sensitive tools to detect early impairments and stratify risk [3,4,5,6,7]. Additionally, the assessment of flow, specifically stroke volume indexed to body surface area (stroke volume index (SVi)), is crucial: a low SVi value (<35 mL/m^2^) may indicate a “paradoxical low-flow” form of AS, even in moderate stenosis [8]. Moderate AS is a condition often understudied compared to severe AS.

Cardiopulmonary exercise testing (CPET) is a cornerstone for assessing functional capacity in heart failure (HF) and valvular diseases, providing objective metrics beyond resting echocardiography. In moderate AS, stratifying patients into low and good functional groups may identify those at higher risk, guiding clinical management [9]. Peak oxygen consumption (VO_2_) is the most recognized functional parameter [10,11]. However, peak VO_2_ is effort-dependent and influenced by non-cardiac factors such as age, skeletal muscle deconditioning and obesity, limiting its specificity in moderate AS with preserved EF. Absolute VO_2_ peak can be misleading in older adults unconditioned who naturally have lower values. In this study, we selected % predicted VO_2_ with a threshold of ≥83% to define preserved functional capacity in elderly patients with moderate aortic stenosis (AS) and preserved ejection fraction (EF ≥ 50%), which is in line with literature suggestions [9,12]. The ventilation to carbon dioxide production slope (VE/VCO_2_ slope) measures ventilatory efficiency, capturing HF-specific pathophysiology like ventilation-perfusion mismatch, elevated pulmonary pressures, and chemoreflex hypersensitivity. VE/VCO_2_ slope is effort-independent, submaximal, and has demonstrated superior prognostic value over peak VO_2_ in HF with preserved EF (HFpEF). Value of VE/VCO_2_ slope ≥ 34 has high specificity in severe outcomes, but ≥30 is more sensitive for detecting early inefficiency [12,13,14,15]. In our study, we defined ventilatory inefficiency as a VE/VCO_2_ slope ≥ 30.

Echocardiography, particularly advanced techniques like global longitudinal strain (GLS) and myocardial work (MW), offers insights into subclinical LV dysfunction, which may precede overt heart failure in AS [16,17,18]. Echocardiographic strain analysis using two-dimensional speckle-tracking echocardiography (STE) provides insights into subclinical myocardial deformation across the left atrium (LA), left ventricle (LV), and right ventricle (RV). LAstrain, is a sensitive tool for assessing LA function across its three physiological phases: reservoir (peak atrial longitudinal strain (PALS)), conduit (LA conduit strain (LAScd)), and contractile (LA contractile strain (LA Scr)). Each phase reflects a distinct aspect of LA mechanics. Parameters such as LV global longitudinal strain (GLS), LA reservoir strain (PALS), and RV free wall strain (RV FWS) detect early dysfunction in AS, correlating with symptoms, fibrosis, and prognosis [19,20,21,22]. In the literature and clinical practice, two abbreviations are commonly encountered for Left atrial reservoir strain: LASr and PALS (peak atrial longitudinal strain). The abbreviation “PALS” is frequently utilised in clinical practice. In moderate AS with preserved EF, integrating CPET-derived functional classifications with strain parameters may enhance risk stratification, as reduced functional capacity (low % predicted VO_2_ or poor VE/VCO_2_) is linked to worse cardiac mechanics [23].

Despite these advances, few studies have compared strain parameters in moderate AS patients stratified by both % predicted VO_2_ and VE/VCO_2_ slope. Leveraging CPET phenotyping in HF cohorts, where low functional is associated with progressive echocardiographic worsening, this approach may identify high-risk subgroups in moderate AS [14].

Parameters like LV EF and GLS evaluate LV systolic performance in AS but do not account for afterload, which varies with AS severity and peripheral vascular resistance, nor do they reflect LV myocardial work or oxygen demand. Noninvasive LV myocardial work (MW), a novel method using pressure-strain loops that integrate GLS with afterload estimates (from blood pressure and transvalvular gradients), provides insights into LV energetics, including global work index (GWI; total work during the cardiac cycle), global constructive work (GCW; work contributing to systolic contraction and diastolic relaxation), global wasted work (GWW; inefficient work due to dyssynchrony), and global work efficiency (GWE; percentage of useful work). Low GLS values indicate impaired LV mechanics, while myocardial work indices quantify LV efficiency, providing prognostic value beyond ejection fraction [24]. In severe AS, MW indices like GWI and GCW have shown independent associations with heart failure symptoms and may outperform GLS in reflecting myocardial remodelling [25].

Therefore, the objectives of this study were: (1) to divide patients with moderate AS and preserved EF into groups based on low versus preserved % predicted VO_2_ and good versus poor ventilatory efficiency (VE/VCO_2_ slope); (2) to compare LA, LV, and RV strain parameters across these groups; and (3) to identify which echocardiographic parameters are best predictors for reduced functional capacity. By integrating CPET and STE, this study aims to refine prognostic phenotyping in moderate AS, facilitating risk stratification.

## 2. Methodology

A total of 116 patients were screened from the outpatient clinic of the Cardiology Department at the University Clinical Center of Serbia between July 2024 and June 2025. Participants presented with either no symptoms or equivocal complaints, including mild dyspnea, worsening dyspnea among those with chronic obstructive pulmonary disease (COPD), nonspecific chest pain, or atypical angina and dizziness unrelated to exertion.

All patients fulfilled predefined inclusion criteria for moderate aortic stenosis (AS), as per the European Association of Cardiovascular Imaging (EACVI) guidelines: aortic valve area (AVA) between 1.0 and 1.5 cm^2^, mean transvalvular pressure gradient of 20–40 mmHg, peak aortic jet velocity of 3.0–4.0 m/s, and preserved left ventricular ejection fraction (LVEF ≥ 50%) confirmed by two-dimensional echocardiography.

Exclusion criteria encompassed LVEF < 50%, resting heart rate > 90 beats per minute, significant concomitant valvular pathology (≥moderate aortic regurgitation, ≥moderate mitral stenosis or regurgitation), moderate-to-severe mitral annular calcification, unstable coronary artery disease, known significant epicardial coronary artery disease, other cardiac disorders (e.g., constrictive pericarditis, pulmonary thromboembolism, primary pulmonary hypertension, or high-output heart failure), and major noncardiac conditions impairing functional capacity.

**Figure 1:**
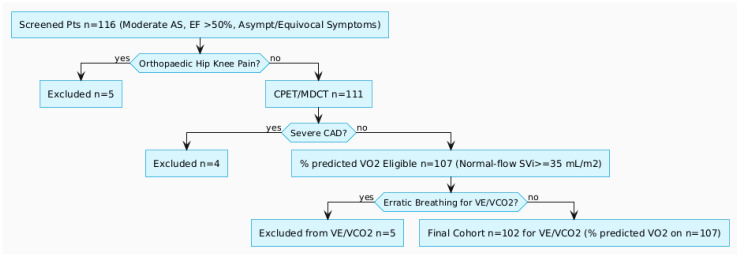
Study flowchart. CPET—cardiopulmonary exercise test, MDCT—multidetector computed tomography, CAD—coronary artery disease.

Of the screened cohort, five patients were excluded due to orthopedic limitations (knee or hip pain) preventing cardiopulmonary exercise testing (CPET). An additional four were excluded post-CPET or multidetector computed tomography (MDCT) due to newly identified severe coronary artery disease. This yielded 107 patients eligible for predicted peak oxygen uptake (VO_2_) assessment. However, five more were excluded from ventilatory efficiency (VE/VCO_2_) analysis owing to erratic breathing patterns, resulting in a final analytical cohort of 102 patients for VE/VCO_2_ analysis. All included patients exhibited normal-flow physiology, with stroke volume index (SVi) ≥ 35 mL/m^2^; no instances of low-flow, low-gradient, or paradoxical low-flow AS were observed (Figure 1).

### 2.1. Echocardiography

All selected patients underwent a standard comprehensive echocardiographic study using a Vivid E95 imaging device (GE Healthcare, Chicago, IL, USA) and stored on a workstation for off-line analysis (EchoPAC, GE Healthcare), including M-mode, 2D echocardiogram, Doppler, tissue Doppler, and multiple transducer positions to record aortic valve jet velocity. Aortic valve area (AVA) was calculated using the continuity equation as per EACVI guidelines. The following parameters were measured offline: left ventricular (LV) wall thickness, volumes, and ejection fraction (EF). E-wave velocity, A-wave velocity, and septal and lateral e’ velocities were measured from mitral valve inflow and mitral annulus tissue Doppler. Tricuspid lateral annulus S’ were measured from tissue Doppler. Right ventricular systolic pressure (RVSP) was derived as the sum of the tricuspid regurgitation jet peak velocity and the estimated mean right atrial pressure based on the diameter and collapsibility of the inferior vena cava. Mitral regurgitation and tricuspid regurgitation were defined as significant when graded as moderate or severe according to current recommendations. LV stroke volume (SV) was derived from the left ventricular outflow tract (LVOT). The biplane Simpson’s method was used to measure LV and left atrial (LA) volumes, indexed to body surface area (BSA).

Offline 2D speckle tracking strain analysis was performed to measure left atrial (LA) and left ventricular (LV) longitudinal strain on the same image and cardiac cycle to eliminate beat-to-beat variability. Analyzed image frame rates were ≥60 frames/s. LA cardiac cycles were defined as: (i) reservoir phase: from ventricular end-diastole until mitral valve opening; (ii) conduit phase: from mitral valve opening through diastasis until the onset of atrial contraction; and (iii) contractile phase: from the onset of atrial contraction until the end of ventricular diastole. Endocardial border tracing was performed automatically, and segments with persistently inadequate tracking after manual adjustment were excluded. LV myocardial systolic function and LA phasic function were studied on apical views. The following myocardial and chamber function values were recorded: LV global longitudinal peak systolic strain (GLS), LA reservoir (PALS), LA contractile and conduit strains (LA Sct and LA Scd, respectively), and LA biplane EF. The time to peak strain (PSD) for both chambers was also recorded. Strain analysis was feasible in all patients included. All echocardiographic and myocardial work analyses were performed by a single experienced echocardiographer (>15 years’ experience), blinded to clinical and CPET data, to minimize variability.

Myocardial work indices were derived using proprietary software (EchoPAC version 204) that integrates LV GLS with blood pressure recordings to construct pressure–strain loops over cardiac cycles. LV GLS was measured from the apical four-, two-, and three- or five-chamber views by tracing the endocardial border at an end-systolic frame, after which the software automatically defined a region of interest, manually corrected to include the entire myocardial thickness when needed. After GLS measurement, the timing of aortic and mitral valve opening and closure, and LV systolic blood pressure were entered into the software. LV systolic pressure was estimated by the sum of the mean aortic transvalvular gradient and non-invasively measured systolic pressure to correct for afterload, as previously described and validated. The software provided four indices of global myocardial work: (i) Left ventricular global work index (LV GWI), calculated as the area within the pressure–strain loop from mitral valve closure to opening; (ii) Left ventricular global constructive work (LV GCW), defined as shortening during systole and lengthening during relaxation; (iii) Left ventricular global wasted work (LV GWW), determined as lengthening during systole and shortening during relaxation; and (iv) Left ventricular global work efficiency (LV GWE), calculated by dividing LV GCW by the sum of LV GCW and LV GWW. All readings were performed by one experienced echocardiographer.

For RV strain measurement, RV-focused apical four-chamber views were acquired in the left lateral position, ensuring no RV foreshortening. Frame rates were set at 50–90 fps, optimized for endocardial definition, with 3–5 cardiac cycles recorded, ECG-gated, and timed to pulmonary valve closure. In the end-diastolic frame, points were placed at the basal septum, basal RV free wall, and apex. The endocardial border was traced semi-automatically, covering the RV free wall and septum, with ROI width adjusted to myocardial thickness. Tracking quality was verified, and poor segments were adjusted or excluded. Three segments (basal, mid, apical for RV free wall) were analyzed with RV free wall strain (FWS) as the average. TAPSE was automatically derived from the longitudinal displacement of the basal RV free wall segment during systole using the speckle-tracking software (EchoPAC, GE Healthcare, version 204).

### 2.2. CPET

The cardiopulmonary exercise test (CPET) was performed using a Schiller CS-200 cycle ergometer (Schiller, CARDIOVIT CS-200 Excellence, Germany) and the R15 ramp protocol, with continuous breath-by-breath analysis of expired gases collected through a well-sealed facemask. CPET data were acquired on a breath-by-breath basis and averaged over 8–10-s intervals to reduce short-term variability. All tests underwent visual quality control, and patients with erratic breathing patterns or poor signal stability were excluded from the analysis.

Throughout the CPET, a 12-lead ECG was continuously recorded, and ventilatory gas exchange variables were measured in real-time, including oxygen uptake (VO_2_) at the first anaerobic threshold (AT), identified using the V-slope method and peak oxygen consumption (peak VO_2_), representing the subject’s maximal aerobic capacity. Additional ventilatory parameters, such as minute ventilation (VE) and breathing reserve, were monitored to evaluate ventilatory efficiency and potential respiratory limitations. Blood pressure and heart rate were measured at baseline, at the end of each workload stage, and during the recovery phase. No participant exhibited electrocardiographic or clinical signs suggestive of myocardial ischemia during or after testing. Tests were terminated upon symptom onset, abnormal hemodynamics, or achievement of RER > 1.05, per safety guidelines for older cardiac patients. All participants completed graded cycle ergometer exercise testing until they reached the point of volitional exhaustion. Submaximal effort was expected in some participants due to the advanced age of the study cohort, the presence of comorbidities and factors such as reduced motivation or lack of familiarity with the CPET procedure. Peak oxygen uptake was determined as the maximum value recorded in the final 30 s of the exercise test, reported as absolute VO_2_ peak (mL/min), adjusted VO_2_ peak (mL/kg/min), or % predicted VO_2_ (% of predicted value based on age, sex, and weight). Exercise ventilation efficiency was addressed by the VE increase for a given VCO_2_ slope and calculated.

Patients were classified according to functional capacity and ventilatory efficiency. Functional capacity was defined by % predicted peak VO_2_, with values ≥ 83% indicating preserved capacity and <83% indicating reduced capacity [12]. Ventilatory efficiency was defined by VE/VCO_2_ slope, with values < 30 considered good and ≥30 considered poor [26,27].

This study was conducted in accordance with the Declaration of Helsinki and approved by the Ethics Committee of the University Clinical Center of Serbia and the Faculty of Medicine, University of Belgrade (protocol code 25/XI-4, 20 November 2024).

## 3. Statistics

Data are expressed as mean ± standard deviation (SD) or percentages unless otherwise specified. Group differences were evaluated using the Student *t* test for normally distributed continuous variables, Mann-Whitney U tests for non–normally distributed continuous variables, and the chi-square or Fisher exact tests for categorical variables. Pearson or Spearman correlation coefficients were used to examine the relationship between continuous variables. To identify independent predictors of a positive exercise test, logistic multivariate analysis was performed, with significant variables included in the statistical model. A *p*-value < 0.05 was considered statistically significant. Associations between CPET and echocardiographic parameters were determined using logistic regression analysis. Only variables that were significantly correlated with the outcome (% predicted VO_2_ < 83% or VE/VCO_2_ slope ≥ 30%) in univariate analysis were considered for entry into the logistic regression model using the Forward LR method, which retained only the most significant predictors based on likelihood ratio tests (*p* < 0.05 for entry, *p* < 0.10 for retention). Data were analyzed using SPSS version 23.

To identify independent predictors of reduced exercise capacity (% predicted VO_2_ < 83%) or poor ventilatory efficiency (VE/VCO_2_ slope ≥ 30%), logistic regression models were constructed. Candidate covariates were selected based on clinical relevance (e.g., age, sex, BMI, β-blocker therapy, AF, COPD, CKD, SBP) and univariate significance (*p* < 0.10). The primary model used forward stepwise selection (likelihood ratio method; *p* < 0.05 for entry, *p* < 0.10 for retention), adhering to the events-per-variable (EPV) rule (≤1 predictor per 10 events; max 5 predictors for ~50 events).

Collinearity was assessed via variance inflation factors (VIF; cutoff > 3 for exclusion) and correlation matrices (Pearson/Spearman |r| > 0.7, with exclusion/replacement of highly correlated variables). Model fit was evaluated using the Hosmer-Lemeshow test (*p* > 0.05) and ROC curve analysis (AUC with 95% CIs). A sensitivity analysis employed the Enter method, forcing inclusion of key covariates, with internal validation via bootstrap resampling (1000 iterations) for optimism-corrected coefficients and 95% CIs.

To address multiple testing across echocardiographic and myocardial work parameters, Benjamini-Hochberg false discovery rate (FDR) correction was applied (q < 0.05 for significance).

## 4. Results

### 4.1. Patient Characteristics

The study included 107 patients with moderate aortic stenosis (AS) and preserved ejection fraction (EF ≥ 50%), recruited between April 2024 and June 2025. Patients were stratified by functional capacity (% predicted peak VO_2_ < 83%, n = 42; ≥83%, n = 65) and ventilatory efficiency (VE/VCO_2_ slope ≥ 30, n = 41; <30, n = 61). Five patients were excluded from VE/VCO_2_ analysis due to irregular breathing patterns affecting measurement reliability, leaving 102 patients for this comparison.

In the % predicted VO_2_ groups, those with reduced functional capacity (<83%) were more often male (77% vs. 53%, *p* = 0.014), while age, body mass index (BMI), blood pressure, comorbidities, and medical therapy were similar (all *p* > 0.05, Table 1). There was no significant difference in β-blocker therapy between groups. All included patients had acceptable spirometry, and no patient met criteria for moderate or severe obstructive impairment prior to CPET.Echocardiographic and CPET Findings.

Patients with reduced functional capacity (% predicted VO_2_ < 83%) demonstrated larger LV end-systolic volumes (43.6 ± 13.4 vs. 38.5 ± 11.3 mL, *p* = 0.035), smaller indexed aortic valve area (0.61 ± 0.09 vs. 0.67 ± 0.14 cm^2^/m^2^, *p* = 0.025), and lower stroke volume index (47.4 ± 8.4 vs. 51.7 ± 9.4 mL/m^2^, *p* = 0.020) compared with those with preserved capacity. LV end-diastolic volume (*p* = 0.055) and global work index (*p* = 0.068) showed trends toward significance. Other strain parameters, including GLS, LA strain, and RV free wall strain, did not differ significantly between groups (Table 2).

In the VE/VCO_2_ groups, patients with a slope ≥ 30 were older (73.9 ± 6.5 vs. 70.2 ± 7.4 years, *p* = 0.011), but no other baseline characteristics differed significantly (Table 3).

By contrast, ventilatory inefficiency (VE/VCO_2_ slope ≥ 30) was associated with more pronounced myocardial dysfunction. These patients had worse LV GLS (−15.8 ± 3.1% vs. −17.6 ± 2.6%, *p* = 0.002), lower TAPSE (16.1 ± 4.4 vs. 18.8 ± 4.8 mm, *p* = 0.005), reduced LA reservoir strain (21.6 ± 8.0% vs. 25.6 ± 8.6%, *p* = 0.019), and impaired LA conduit strain (−8.4 ± 5.1% vs. −12.7 ± 5.3%, *p* < 0.001). RV free wall strain was also reduced (−19.5 ± 5.6% vs. −22.0 ± 5.8%, *p* = 0.029). Myocardial work indices were significantly altered, with lower GWI and GCW, higher GWW, and reduced GWE (all *p* < 0.05). Additionally, patients with VE/VCO_2_ ≥30 had smaller AVA (1.16 ± 0.17 vs. 1.26 ± 0.18 cm^2^, *p* = 0.010) and higher mean pressure gradients (26.2 ± 5.8 vs. 24.0 ± 4.9 mmHg, *p* = 0.043) (Table 4).

The correlation between left ventricular global longitudinal strain (GLS) and VE/VCO_2_ slope is illustrated via scatterplot in Supplementary Materials Figure S2. Likewise, the negative association between global work index (GWI) and VE/VCO_2_ slope is depicted in Supplementary Materials Figure S3.

Because multiple echocardiographic and myocardial work parameters were tested, we applied false discovery rate (FDR) correction to minimize the risk of type I error. The main associations remained significant after correction, supporting the robustness of the findings and reducing the likelihood that they are driven by multiple comparisons.

**Figure 2:**
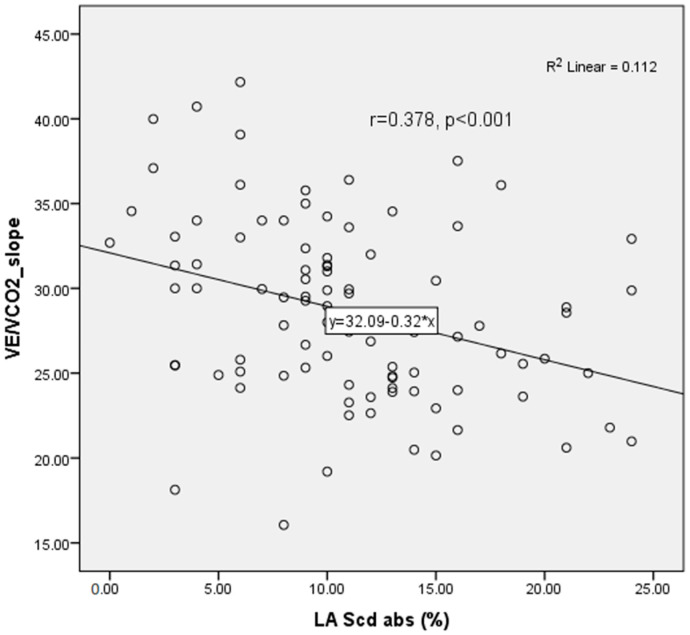
Scatter plot and Spearman correlation of LA Scd % and VE/VCO_2_ Slope in the study population.

LAScd showed a significant inverse correlation with the VE/VCO_2_ slope (r = 0.378, *p* < 0.001), consistent with impaired left atrial–pulmonary coupling in patients with reduced ventilatory efficiency (Figure 2).

Receiver operating characteristic (ROC) analysis was performed to assess the ability of LAScd to identify patients with ventilatory inefficiency (VE/VCO_2_ ≥ 30). The area under the curve (AUC) was 0.723 (95% CI 0.623–0.823, *p* < 0.001), indicating fair discrimination (Supplementary Materials Figure S1).

To evaluate threshold robustness, the analysis was repeated using a VE/VCO_2_ slope ≥ 34 to define ventilatory inefficiency. In this model, only TAPSE remained independently associated with the outcome (B = −1.76, SE = 0.62, *p* = 0.005, OR = 0.17 (95% CI not computed by SPSS for bootstrap model)), while the direction of associations for other variables remained consistent but no longer reached statistical significance. These results suggest that right ventricular function retains prognostic relevance even when a stricter criterion for ventilatory inefficiency is applied.

### 4.2. Univariate and Multivariate Analyses

In univariate analysis, % predicted VO_2_ correlated positively with age, AVAi, stroke volume index, and absolute value of RV free wall strain. These findings indicate that forward flow reserve, rather than subclinical LV dysfunction, is the principal determinant of aerobic capacity in moderate AS. Although SVi and AVAi both showed moderate correlations with exercise performance, neither variable remained an independent predictor in multivariable modeling.

In contrast, VE/VCO_2_ slope showed stronger and more consistent associations with indices of myocardial mechanics. Age and systolic blood pressure correlated modestly with ventilatory efficiency, but echocardiographic parameters demonstrated the most robust relationships. GLS, GWI, GCW, LA Scd, and TAPSE were all significantly associated with VE/VCO_2_ slope, highlighting the integrated contribution of LV, LA, and RV mechanics to ventilatory control. Among these, LA Scd emerged as the only independent predictor in multivariable analysis (Table 5). This underscores the pivotal role of atrial conduit function in determining ventilatory efficiency, likely through its impact on LV filling pressures, pulmonary vascular load, and RV–pulmonary coupling.

Internal validation with 1000 bootstrap samples showed overall model stability and low coefficient bias. The optimism-corrected regression coefficients (B (95% CI)) were: age 0.047 (−0.043 to 0.178), GLS 0.361 (−0.114 to 1.026), TAPSE −0.421 (−1.903 to 0.961), LAScd 0.131 (0.025 to 0.309), GWI −0.001 (−0.006 to 0.005), GCW 0.003 (−0.002 to 0.008], and systolic blood pressure (SBP) −0.045 (−0.120 to −0.004). Among these, LAScd (*p* = 0.019) and SBP (*p* = 0.083) remained significant or near-significant after correction, confirming limited overfitting and good internal consistency of the final model (Supplementary Materials Table S1).

In the sensitivity multivariable model incorporating prespecified clinical covariates (age, sex, BMI, β-blocker therapy, AF, COPD, CKD, and SBP) irrespective of univariable significance, associations aligned closely with the main analysis. Following 1000 bootstrap resamples, optimism-corrected coefficients (B [95% CI]) included: age 0.043 (−0.071–0.212), GLS 0.269 (−0.359–1.336), TAPSE −0.769 (−3.042–0.995), LAScd 0.160 (0.054–0.521), GWI −0.002 (−0.011–0.008), GCW 0.003 (−0.003–0.012), SBP−0.052 (−0.186–0.000), BMI 0.046 (−0.116–0.257), and COPD −20.661 (−23.281–−17.539). Among these, LAScd (*p* = 0.024) and COPD (*p* = 0.003) remained independently associated with VE/VCO_2_ slope ≥ 30, affirming model robustness against confounders and minimal overfitting post-validation. The markedly large negative coefficient for COPD (B = −20.661, *p* = 0.003), although statistically significant, is attributable to its rarity, coded dichotomously as yes/no for diagnosis, and quasi-complete separation—stemming from the pre-CPET spirometry exclusion of moderate-to-severe cases, which yielded few or no instances of ventilatory inefficiency among affected individuals and thus unstable parameter estimates. Nonetheless, this underscores the primary model’s resilience to confounding and minimal overfitting after validation, with COPD’s association warranting cautious interpretation.

## 5. Discussion

### 5.1. Main Findings

In this prospective cohort, 40% of patients with moderate AS and preserved EF exhibited reduced exercise capacity, and 40% demonstrated ventilatory inefficiency. Severity of AS and comorbidity burden were less influential than underlying myocardial dysfunction. Strain-derived indices—including LV GLS, LA reservoir and conduit strain, and RV free wall strain—were strongly associated with ventilatory inefficiency but not with exercise capacity. By contrast, exercise capacity was linked to forward flow measures (stroke volume index, AVAi). Notably, LA conduit strain emerged as the strongest independent predictor of ventilatory inefficiency.

### 5.2. Underlying Mechanisms and Pathophysiology

Moderate AS is increasingly recognized as a heterogeneous disease, with variable patterns of LV remodeling and atrial–ventricular interaction that explain differences in clinical outcomes [28,29].

Accurate risk stratification is critical, as many patients with preserved EF harbour subclinical myocardial damage not evident on resting echocardiography. Functional testing offers an integrated assessment of cardiovascular reserve that complements structural imaging.

Our data show that impaired forward flow (SVi, AVAi) primarily determines exercise capacity, whereas ventilatory inefficiency reflects a more global cardiopulmonary burden. The strong link between LA conduit strain and ventilatory inefficiency highlights the pathophysiological role of atrial function in LV filling and pulmonary vascular load. Impaired LA conduit function elevates filling pressures, increases pulmonary resistance, and predisposes to RV dysfunction, all of which worsen ventilatory efficiency. This aligns with prior studies demonstrating that LA strain, particularly the conduit component, is a sensitive marker of diastolic dysfunction and pulmonary hypertension, and correlates with reduced exercise tolerance in both HF and valvular disease [30,31].

### 5.3. Clinical Implications

Risk stratification in moderate AS remains a pressing challenge. Conventional echocardiography often underestimates disease burden in patients with preserved EF, while functional assessment via CPET provides objective, integrated measures of reserve. In our study, VE/VCO_2_ slope ≥ 30 was a more robust marker of subclinical dysfunction than % predicted VO_2_, echoing prior evidence from HF and AS cohorts [30]. Resting echocardiography often fails to detect these abnormalities, whereas functional assessment provides an objective and integrated measure of cardiovascular reserve.

Gherbesi et al. reviewed the physical basis and clinical applications of speckle-tracking echocardiography, emphasizing its role in assessing left ventricular function [32].

Our findings align with recent research highlighting the importance of left atrial (LA) function in aortic stenosis (AS). Specifically, Tan et al. demonstrated the prognostic value of LA strain in AS using a competing risk analysis, further solidifying the role of LA strain as a valuable marker for risk stratification [33].

Beyond LA metrics, right heart strain also offers robust prognostic value: a meta-analysis establishes a substantial association between RV FWS and adverse outcomes (e.g., HF hospitalisation, mortality) in the AS population, independent of LV function and AS severity. This incremental utility enhances multiparametric models for early decompensation detection [34]. Clinically, STE integration could refine early TAVR: in equivocal moderate AS, LA/RV/RA strain abnormalities flag high-risk phenotypes for timely intervention, mitigating ischemia and optimizing recovery [35]. Serial STE/CPET at 6–12 months could detect early all-chamber dysfunction and guide interventions in moderate AS.

Importantly, ongoing trials are investigating whether earlier intervention benefits selected patients with moderate AS. TAVR-UNLOAD reported improved quality of life without mortality benefit, while PROGRESS and EXPAND TAVR II are evaluating early TAVR in high-risk phenotypes [36,37].

Our findings suggest that patients with impaired ventilatory efficiency and abnormal LA conduit strain may represent one such high-risk subgroup warranting closer surveillance and possibly earlier intervention [31,38].

### 5.4. The Role of LA Conduit Strain

Among strain parameters, LA conduit strain was the only independent predictor of ventilatory inefficiency. This reflects its integrative role in preload reserve, LV filling, and pulmonary vascular coupling. Beyond mechanics, impaired LA conduit strain serves as a sensitive marker of cardiomyopathy severity, capturing subclinical dysfunction not visible with traditional measures. Prior studies in AS and cardiomyopathy support its prognostic value, with LAScd consistently associated with adverse outcomes, atrial fibrosis, and impaired remodelling [33,39,40,41].

In ROC analysis, LAScd demonstrated fair discriminatory power for identifying ventilatory inefficiency. This supports its physiological relevance as a marker of impaired atrial–pulmonary coupling in moderate AS. Although discrimination was moderate, this finding reinforces the concept that LA conduit function reflects early hemodynamic adaptation in this population. External validation in larger cohorts is needed to confirm these results and establish clinical cut-off values. Calibration plots and decision-curve analyses were not performed; clinical utility requires further testing.

## 6. Limitations

This study has several limitations. First, the sample size discrepancy between % predicted VO_2_ groups (n = 107) and VE/VCO_2_ groups (n = 102, due to the exclusion of five patients with irregular breathing) suggests potential data inconsistencies, which may affect generalizability. Second, the modest sample size may limit statistical power for detecting differences, such as GLS in the % predicted VO2 groups. Third, the forward likelihood ratio (LR) method in multivariate analysis may have excluded relevant predictors due to its data-driven approach, and a larger sample could enable more robust modeling. Fifth, the study did not assess long-term outcomes (e.g., mortality, heart failure hospitalization), limiting prognostic conclusions. Finally, the single-center design and exclusion of patients with significant comorbidities may reduce generalizability.

Reproducibility of advanced imaging (e.g., GLS, LAScd, MW) relies on single-observer analysis here, without ICC reporting. While blinding reduced bias, unassessed intra-/inter-observer variability introduces potential measurement error, warranting multi-observer validation in future studies.

We acknowledge that ventilatory and chronotropic factors may influence exercise capacity and ventilatory efficiency. Although β-blocker therapy was comparable between groups, spirometry was performed in all patients with exclusion of relevant pulmonary impairment, and patients with erratic breathing patterns were excluded from CPET analysis, residual confounding from heart-rate reserve or subtle ventilatory limitation cannot be fully excluded.

The inclusion of clinically relevant confounders in the sensitivity model confirmed that the observed associations were not driven by differences in baseline therapy or comorbidities. Nevertheless, although the model was internally validated by bootstrap resampling, external validation in an independent population was not possible. Thus, while the results appear stable, residual confounding and limited generalizability cannot be completely excluded.

The absence of explicit low-flow stratification (all SVi ≥35 mL/m^2^) limits applicability to low-flow cases.

## 7. Conclusions

This study demonstrates that ventilatory inefficiency is more closely associated with subclinical LV, LA, and RV dysfunction than with exercise capacity in patients with moderate aortic stenosis and preserved ejection fraction. LA conduit strain (LAScd) emerged as the strongest independent predictor, underscoring its potential mechanistic role in atrial–pulmonary coupling and its value as a sensitive marker of early hemodynamic impairment. These findings are exploratory and hypothesis-generating; they suggest that combined CPET and strain analysis may improve physiological characterisation and identify patients who could benefit from closer follow-up. However, the absence of longitudinal or outcome data precludes conclusions regarding prognosis or treatment effects.

## Figures and Tables

**Table 1:** Presents the clinical characteristics and treatment distribution for the overall study population (n = 107) and the two subgroups stratified by percentage of predicted VO_2_.

	All (n = 107)	% Predicted VO_2_ ≥ 83% (n = 65)	% Predicted VO_2_ ˂ 83% (n = 42)	*p* Value
Age, y	71.68 ± 7.36	72.2 ± 7.21	70.71 ± 7.40	0.372
Male sex %	62	53	77	**0.014**
Body mass index, kg/m^2^	27.53 ± 3.46	27.53 ± 3.62	27.72 ± 3.20	0.780
Systolic blood pressure, mm Hg	132.59 ± 16.64	131.72 ± 17.19	132.72 ± 16.11	0.775
Hypertension, %	86	82	92	0.139
Diabetes mellitus, %	27	26	29	0.452
Dyslipidemia, %	57	63	47	0.094
Atrial fibrillation, %	15	14	16	0.539
COPD, %	7	6	8	0.539
CKD, %	11	10	12	0.472
Therapy				
ACE inhibitor, %	61	58	64	0.359
ARB, %	16	18	13	0.331
CCB, %	44	42	49	0.314
Beta-blocker, %	71	65	80	0.092
Statin, %	64	62	67	0.387
SGLT2 inhibitor, %	15	13	18	0.363
Diuretic, %	61	60	62	0.524

BMI, body mass index; COPD, chronic obstructive pulmonary disease; CKD, chronic kidney disease; ARB, angiotensin II receptor blocker; CCB, calcium channel blocker.

**Table 2:** Echocardiographic Variables for the overall study population (n = 107) and the two subgroups stratified by percentage of predicted VO_2_.

	All (n = 107)	% Predicted VO_2_ ≥ 83% (n = 65)	% Predicted VO_2_ < 83% (n = 42)	*p* Value
Basic Echocardiographic Parameters				
LV EDV, mL	97.62 ± 26.17	94.14 ± 25.37	104.02 ± 26.41	0.055
LV ESV, mL	40.39 ± 12.34	38.51 ± 11.26	43.64 ± 13.38	**0.035**
LV EF, %	58.76 ± 5.11	59.12 ± 5.38	58.31 ± 4.76	0.426
E/e’ Ratio	14.72 ± 5.97	14.11 ± 5.44	15.71 ± 6.74	0.178
LAVI, mL/m^2^	38.80 ± 8.72	38.74 ± 8.78	38.91 ± 8.52	0.923
RVSP, mmHg	31.90 ± 6.22	31.54 ± 6.07	32.55 ± 6.70	0.483
TAPSE, mm	17.54 ± 4.92	18.52 ± 4.25	16.21 ± 5.59	**0.017**
Aortic PG, mmHg	42.27 ± 8.67	42.75 ± 9.24	41.49 ± 7.75	0.467
Aortic MPG, mmHg	24.66 ± 5.27	24.92 ± 5.54	24.22 ± 4.91	0.508
AVA, cm^2^	1.22 ± 0.18	1.23 ± 0.18	1.22 ± 0.17	0.781
AVAi, cm^2^/m^2^	0.65 ± 0.12	0.67 ± 0.14	0.61 ± 0.09	**0.025**
SVi, mL/m^2^	50.04 ± 9.17	51.65 ± 9.39	47.39 ± 8.35	**0.020**
S’ TV, cm/s	11.92 ± 2.41	12.02 ± 2.42	11.81 ± 2.46	0.671
LVmass indexed, g/m^2^	107.41 ± 24.00	109.25 ± 23.41	105.11 ± 25.10	0.394
Strain and Myocardial Work Parameters				
GLS, %	−16.83 ± 2.93	−17.18 ± 3.08	−16.33 ± 2.68	0.144
PSD, ms	66.76 ± 28.81	67.88 ± 27.78	65.36 ± 30.79	0.662
PALS, %	23.64 ± 8.56	24.69 ± 7.83	22.41 ± 9.51	0.178
LA Scd, %	−10.80 ± 5.49	−11.23 ± 5.09	−10.26 ± 6.06	0.375
LA Sct, %	−12.89 ± 6.67	−13.52 ± 6.23	−12.17 ± 7.17	0.302
EF LA, %	48.73 ± 14.20	49.92 ± 12.61	47.91 ± 15.96	0.469
RV FWS, %	−20.61 ± 5.90	−21.45 ± 4.77	−19.34 ± 7.28	0.073
GWI, mmHg%	1975.00 ± 470.51	2045.20 ± 486.40	1874.12 ± 440.32	0.068
GCW, mmHg%	2415.53 ± 476.37	2473.09 ± 506.28	2334.64 ± 429.20	0.146
GWW, mmHg%	203.55 ± 118.02	203.49 ± 107.00	199.93 ± 128.56	0.877
GWE, %	90.56 ± 5.35	90.80 ± 5.08	90.26 ± 5.70	0.611

LV, left ventricle; LA, left atrium; RV, right ventricle; EDV, end-diastolic volume; ESV, end-systolic volume; EF, ejection fraction; E/e’, ratio of mitral peak velocity of early filling to early diastolic mitral annular velocity; LAVI, left atrial volume index; RVSP, right ventricular systolic pressure; TAPSE, tricuspid annular plane systolic excursion; PG, peak gradient; MPG, mean pressure gradient; AVA, aortic valve area; AVAi, indexed aortic valve area; S’ TV, tricuspid lateral annulus S’; LVmass BSA, left ventricular mass indexed to body surface area; SVi, strike volume indexed to body surface area, GLS, global longitudinal strain; PSD, post-systolic dispersion; PALS, peak atrial longitudinal strain; LA Scd, LA conduit strain velocity during diastole; LA Sct, LA contractile strain; EF LA, left atrial ejection fraction; RV FWS, right ventricle free wall strain; GWI, global work index; GCW, global constructive work; GWW, global wasted work; GWE, global work efficiency.

**Table 3:** Presents the clinical characteristics and treatment distribution for the overall study population (n = 102) and the two subgroups stratified by ventilatory efficiency.

	All (n = 102)	VE/VCO_2_ ≥ 30% (n = 41)	VE/VCO_2_ ˂ 30% (n = 61)	*p* Value
Age, y	71.72 ± 7.27	73.93 ± 6.47	70.23 ± 7.43	**0.011**
Male sex %	63	56	67	0.299
BMI, kg/m^2^	27.68 ± 3.40	28.09 ± 3.66	27.42 ± 3.21	0.331
Systolic blood pressure, mm Hg	132.67 ± 16.59	131.27 ± 15.65	133.61 ± 17.25	0.488
Hypertension, %	86	80	90	0.242
Diabetes mellitus, %	27	29	25	0.653
Dyslipidemia, %	56	54	58	0.686
Atrial fibrillation, %	15	15	15	1.000
COPD, %	7	7	7	1.000
CKD, %	11	17	7	0.094
Therapy				
ACE inhibitor, %	61	58	63	0.676
ARB, %	16	15	17	1.000
CCB, %	45	43	47	0.838
Beta-blocker, %	70	62	75	0.191
Statin, %	63	57	67	0.401
SGLT2 inhibitor, %	15	18	13	0.580
Diuretic, %	61	60	62	1.000

BMI, body mass index; COPD, chronic obstructive pulmonary disease; CKD, chronic kidney disease; ARB, angiotensin II receptor blocker; CCB, calcium channel blocker.

**Table 4:** Echocardiographic Variables for the overall study population (n = 102) and the two subgroups stratified by ventilatory efficiency.

	All (n = 102)	VE/VCO_2_ ≥ 30% (n = 41)	VE/VCO_2_ < 30% (n = 61)	*p* Value
Basic Echocardiographic Parameters				
LV EDV, mL	97.52 ± 26.42	92.00 ± 23.89	101.23 ± 27.57	0.084
LV ESV, mL	40.42 ± 12.30	41.79 ± 12.81	38.39 ± 11.34	0.173
LV EF, %	58.70 ± 4.98	58.80 ± 4.63	58.54 ± 5.53	0.792
E/e’ Ratio	14.79 ± 6.12	15.09 ± 7.26	14.35 ± 3.91	0.550
LAVI, mL/m^2^	38.79 ± 8.84	39.57 ± 9.04	37.63 ± 8.51	0.279
RVSP, mmHg	31.98 ± 6.14	31.98 ± 6.15	31.97 ± 6.21	0.994
TAPSE, mm	17.68 ± 4.82	16.05 ± 4.38	18.77 ± 4.82	**0.005**
Aortic PG, mmHg	42.58 ± 8.77	43.98 ± 9.93	41.64 ± 7.84	0.188
Aortic MPG, mmHg	24.84 ± 5.34	26.15 ± 5.77	23.97 ± 4.88	**0.043**
AVA, cm^2^	1.22 ± 0.18	1.16 ± 0.17	1.26 ± 0.18	**0.010**
AVAi, cm^2^/m^2^	0.65 ± 0.13	0.63 ± 0.16	0.66 ± 0.10	0.192
SVi, mL/m^2^	50.16 ± 9.19	47.88 ± 9.32	51.66 ± 8.86	**0.043**
S’ TV, cm/s	11.95 ± 2.44	11.70 ± 2.63	12.12 ± 2.32	0.407
LVmass indexed, g/m^2^	107.32 ± 24.11	109.37 ± 24.55	105.89 ± 23.91	0.481
Strain and Myocardial Work Parameters				
GLS, %	−16.88 ± 2.94	−15.79 ± 3.05	−17.61 ± 2.64	**0.002**
PSD, ms	67.96 ± 29.14	68.72 ± 29.87	67.44 ± 28.88	0.829
PALS, %	24.01 ± 8.53	21.61 ± 7.98	25.62 ± 8.58	**0.019**
LA Scd, %	−10.94 ± 5.58	−8.37 ± 5.05	−12.67 ± 5.27	**0.000**
LA Sct, %	−13.14 ± 6.63	−13.08 ± 7.09	−13.22 ± 5.96	0.919
EF LA, %	49.03 ± 14.08	47.17 ± 14.27	50.28 ± 13.94	0.277
RV FWS, %	−20.99 ± 5.80	−19.47 ± 5.56	−22.02 ± 5.77	**0.029**
GWI, mmHg%	1980.13 ± 472.11	1824.71 ± 521.79	2084.59 ± 407.54	**0.006**
GCW, mmHg%	2422.52 ± 478.89	2289.93 ± 485.73	2511.64 ± 456.80	**0.021**
GWW, mmHg%	207.31 ± 117.27	240.44 ± 149.83	185.05 ± 83.23	**0.019**
GWE, %	90.39 ± 5.32	88.66 ± 6.86	91.56 ± 3.59	**0.006**

LV, left ventricle; LA, left atrium; RV, right ventricle; EDV, end-diastolic volume; ESV, end-systolic volume; EF, ejection fraction; E/e’, ratio of mitral peak velocity of early filling to early diastolic mitral annular velocity; LAVI, left atrial volume index; RVSP, right ventricular systolic pressure; TAPSE, tricuspid annular plane systolic excursion; PG, peak gradient; MPG, mean pressure gradient; AVA, aortic valve area; AVAi, indexed aortic valve area; S’ TV, tricuspid lateral annulus S’; LVmass BSA, left ventricular mass indexed to body surface area; SVi, strike volume indexed to body surface area, GLS, global longitudinal strain; PSD, post-systolic dispersion; PALS, peak atrial longitudinal strain; LA Scd, LA conduit strain velocity during diastole; LA Sct, LA contractile strain; EF LA, left atrial ejection fraction; RV FWS, right ventricle free wall strain; GWI, global work index; GCW, global constructive work; GWW, global wasted work; GWE, global work efficiency.

**Table 5:** Univariate and Multivariate Analysis of % Predicted VO_2_ and VE/VCO_2_ Slope.

	Univariate		Multivariable Model	
Variable	Correlation Coefficient r	*p*-Value	OR (95% CI)	*p*-Value
**% predicted VO_2_**				
**Clinical Parameters**				
Age, y	0.220	0.035		
**Echocardiographic Parameters**				
AVAi, cm^2^/m^2^	0.270	0.009		
RV FWS abs %	0.207	0.045		
SVI, mL	0.245	0.014		
**VE/VCO_2_ slope**				
**Clinical Parameters**				
Age, y	0.238	0.017		
Systolic BP, mmHg	−0.238	0.017		
**Echocardiographic Parameters**				
GLS, abs %	−0.243	0.019		
GWI, mmHg%	−0.309	0.002		
GCW, mmHg%	−0.267	0.004		
LA Scd abs %	−0.378	0.000	1.177 (1.069–1.296)	**0.001**
TAPSE, mm	−0.260	0.012		

## Data Availability

The data presented in this study are available on request from the corresponding author.

## Supplementary Materials

The following supporting information can be downloaded at: https://www.mdpi.com/article/10.3390/jcm14228065/s1, Figure S1: ROC curve regarding diagnostic accuracy of LA conduit strain (LAScd) in predicting patients with ventilatory inefficiency (VE/VCO_2_ ≥30); Figure S2: Scatter plot of GLS % and VE/VCO_2_ Slope in the study population. Spearman correlation r = 0.264, *p* = 0.007; Figure S3: Scatter plot of GWI and VE/VCO_2_ Slope in the study population. Spearman correlation of GWI and VE/VCO_2_ Slope r = −3.08, *p* = 0.001; Table S1: Bootstrap Linear Regression Analysis of Echocardiographic and Clinical Variables.
