# Supplementary material for: Comprehensive Echocardiographic Assessment in Moderate Aortic Stenosis with Preserved Ejection Fraction Using Two-Dimensional Speckle-Tracking Echocardiography: Association with Functional Capacity
Source: J Clin Med. 2025 Nov 14;14(22):8065. doi: 10.3390/jcm14228065 (PMC12653056; doi:10.3390/jcm14228065)
Supplement: Supplementary file 1 [file jcm-14-08065-s001.zip › jcm-3954203-supplementary.pdf]

Supplementary tables and figures

Supplementary figure S1.

ROC curve regarding diagnostic accuracy of LA conduit strain (LAScd) in predicting patients with ventilatory inefficiency ($VE/VCO_2 \geq 30$).

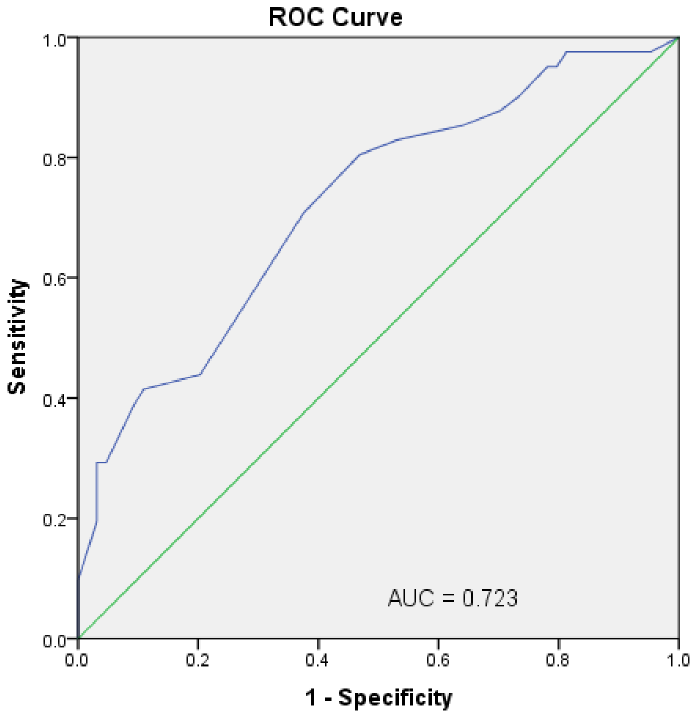

Supplementary figure S2.

Scatter plot of GLS % and VE/VCO2 Slope in the study population. Spearman correlation r=0.264, P=0.007

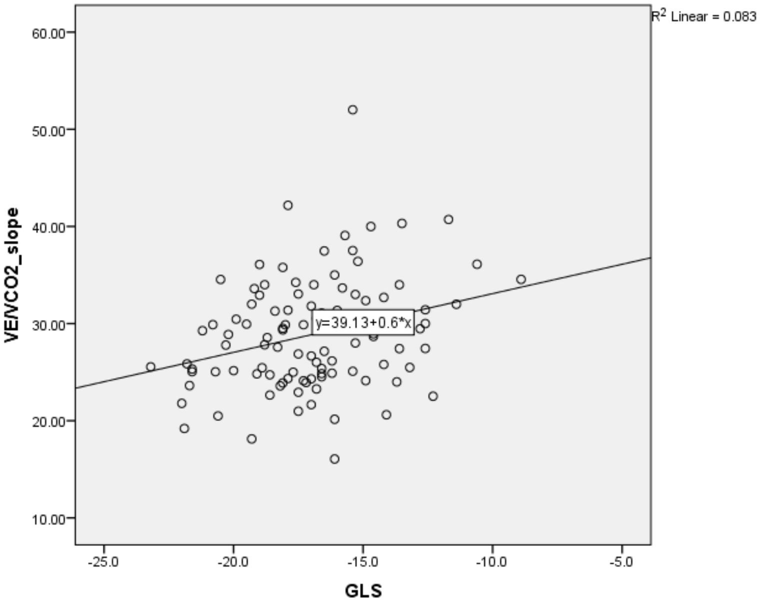

Supplementary figure S3

Scatter plot of GWI and VE/VCO2 Slope in the study population. Spearman correlation of GWI and VE/VCO2 Slope r=-3.08, P=0.001

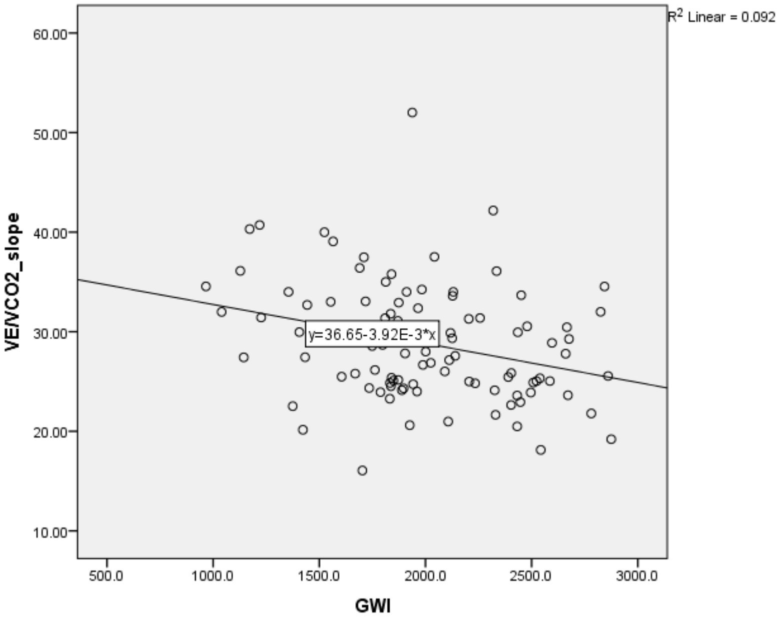

Supplementary Table S1.

Bootstrap Linear Regression Analysis of Echocardiographic and Clinical Variables

Variable	B	Bias	Std. Error	Sig. (2-tailed)	95% CI (Lower–Upper)
Age	0.047	0.009	0.058	0.312	−0.043 – 0.178
GLS	0.361	0.062	0.283	0.137	−0.114 – 1.026
TAPSE	−0.421	−0.034	0.711	0.528	−1.903 – 0.961
LAScd	0.131	0.024	0.070	0.019	0.025 – 0.309
GWI	−0.001	0.000	0.003	0.728	−0.006 – 0.005
GCW	0.003	0.000	0.002	0.196	−0.002 – 0.008
SBP	−0.045	−0.009	0.030	0.083	−0.120 – −0.004
Constant	5.706	0.917	6.171	0.293	−4.886 – 19.759
